# Parameterizing the Transport Pathways for Cell Invasion in Complex Scaffold Architectures

**DOI:** 10.1089/ten.tec.2015.0483

**Published:** 2016-03-21

**Authors:** Jennifer C. Ashworth, Marco Mehr, Paul G. Buxton, Serena M. Best, Ruth E. Cameron

**Affiliations:** ^1^ Department of Materials Science and Metallurgy, University of Cambridge, Cambridge, United Kingdom.; ^2^ Geistlich Pharma AG, Core Technologies, Wolhusen, Switzerland.

## Abstract

Interconnecting pathways through porous tissue engineering scaffolds play a vital role in determining nutrient supply, cell invasion, and tissue ingrowth. However, the global use of the term “interconnectivity” often fails to describe the transport characteristics of these pathways, giving no clear indication of their potential to support tissue synthesis. This article uses new experimental data to provide a critical analysis of reported methods for the description of scaffold transport pathways, ranging from qualitative image analysis to thorough structural parameterization using X-ray Micro-Computed Tomography. In the collagen scaffolds tested in this study, it was found that the proportion of pore space perceived to be accessible dramatically changed depending on the chosen method of analysis. Measurements of % interconnectivity as defined in this manner varied as a function of direction and connection size, and also showed a dependence on measurement length scale. As an alternative, a method for transport pathway parameterization was investigated, using percolation theory to calculate the diameter of the largest sphere that can travel to infinite distance through a scaffold in a specified direction. As proof of principle, this approach was used to investigate the invasion behavior of primary fibroblasts in response to independent changes in pore wall alignment and pore space accessibility, parameterized using the percolation diameter. The result was that both properties played a distinct role in determining fibroblast invasion efficiency. This example therefore demonstrates the potential of the percolation diameter as a method of transport pathway parameterization, to provide key structural criteria for application-based scaffold design.

## Introduction

The physical properties and clinical performance of a tissue engineering scaffold are intimately linked to its porous structure. Understanding the characteristics of this structure is therefore crucial for efficient scaffold design and optimization. Some of these characteristics, such as pore size and porosity, are straightforward to measure using microscopic or tomographic imaging techniques.^1,2^ The presence of transport pathways through the pore space is also vital, for determining permeability,^3^ enhancing cell distribution,^4^ and facilitating cell–cell interactions similar to those found *in vivo*.^5^ However, this aspect of the pore structure, commonly termed the “interconnectivity,” is difficult to define, with interpretations in literature ranging from the accessible pore volume to the number of holes in a pore wall.^6^

The aim of this work is to examine a range of existing methods that fall under the heading of “interconnectivity characterization,” and to assess their relevance for predicting the transport characteristics of the pore space, using an *in vitro* model for cell invasion. We use freeze-dried collagen scaffolds as a model system, since natural polymer scaffolds generally contain greater structural complexity than the more regular, but less biologically active, scaffolds that may be fabricated from synthetic polymers.^7^ This complexity presents particular difficulties when it comes to quantitative description of the transport pathways through the pore space. Apart from testing the applicability of each technique to the complex architecture of collagen scaffolds, we compare the results obtained from each method, identifying the strengths and limitations of each.

Finally, we show the importance of characterizing the availability of transport pathways as a function of direction, by examining fibroblast invasion in response to structural anisotropy as proof of principle. In this way, we demonstrate a method for parameterization of the transport pathways through a scaffold, providing the potential for key structure–function relationships to be identified for enhanced tissue regeneration.

## Materials and Methods

### Scaffold fabrication

Collagen scaffolds were fabricated by freeze-drying a suspension of insoluble fibrillar type I collagen from bovine Achilles tendon (Sigma-Aldrich), as previously described.^8^ Briefly, collagen was added at 1% (w/v) to either 0.05 M acetic acid (Alfa-Aesar) or 0.001 M hydrochloric acid (Sigma-Aldrich), before overnight hydration and subsequent homogenization. The collagen suspensions were cooled at 1.2°C min^−1^ to the freezing temperature of −35°C. After complete freezing, a pressure of 80 mTorr and a temperature of 0°C were maintained for ice sublimation, until all ice had been removed.

The resulting scaffolds were chemically cross-linked using 1-ethyl-3-(3-dimethylaminopropyl) carbodiimide hydrochloride (EDC; Sigma-Aldrich) and *N*-hydroxysuccinimide (NHS; Sigma-Aldrich), with 95% ethanol as solvent. EDC and NHS were used in the molar ratio 5:2:1 relative to the collagen carboxylic acid groups (EDC:NHS:COOH). Scaffolds were immersed in the cross-linking solution for 2 h, before thorough washing with distilled water (5 × 5 min), and drying using the same freeze-drying cycle as before.

### Image acquisition

Scanning Electron Microscopy (SEM) was used for qualitative structural assessment. Scaffolds were sectioned using a scalpel, in the planes perpendicular to and containing the direction of solidification, and mounted on a conducting stub using carbon tape. Samples were then sputter-coated with gold/platinum, and a JEOL JSM-820 system was used for image acquisition, in Secondary Electron mode at 10 kV.

For three-dimensional (3D) visualization by Micro-computed tomography (CT), a Skyscan 1072 system (Bruker) was used to image scaffold samples cut with a 5 mm biopsy punch. Projection images were taken at 25 kV and 138 μA, with 0.23° rotation steps and 7.5 s image acquisition time, averaged over four frames. Magnification was set at 75×, pixel size 3.74 μm. Projections were processed into 3D datasets using the Skyscan reconstruction software NRecon, before transferring to the image processing software Fiji for binarization.^9^ The Trainable Segmentation plugin within Fiji was used to classify image pixels using training examples provided by the user.^10^ Image noise was reduced using individual z-slice despeckle, followed by a 2 × 2 × 2 median filter in 3D. The resulting binary 3D dataset was visualized using the Volume Viewer plugin within the Fiji software.

### Quantitative transport pathway analysis

**FIG. 1.**
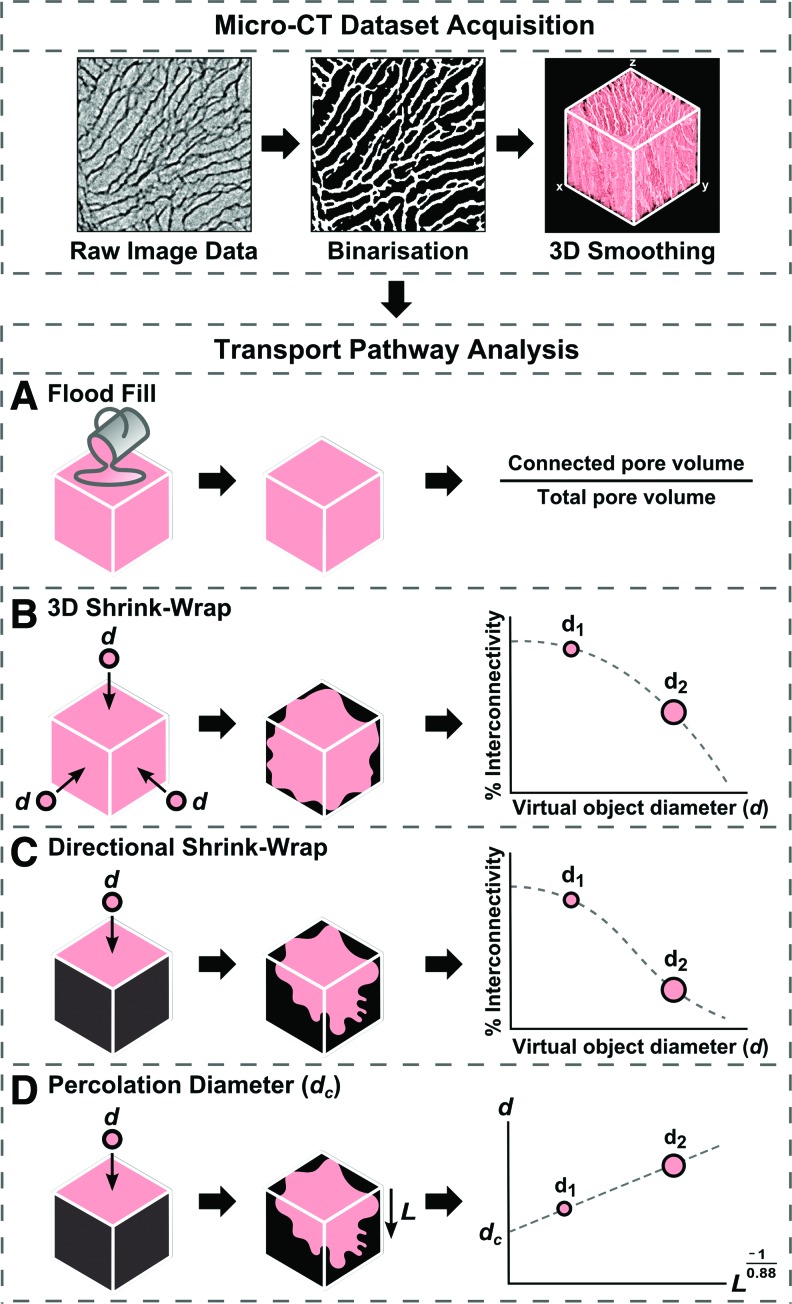
Schematic of the image processing routes investigated for the analysis of scaffold transport characteristics. Method A, Flood Fill, identifies the largest connected group of pore space voxels. Method B, three-dimensional (3D) Shrink-Wrap, identifies the accessible volume for a virtual object of defined diameter, traveling from the outside of the dataset. Method C, Directional Shrink-Wrap, is a variation of Method B, in which the virtual object can only access the scaffold interior from one surface. Method D, Percolation Diameter, describes the largest sphere that can travel to infinite distance through the scaffold in a direction of interest, calculated by measurement of accessible distance as a function of virtual object diameter. Color images available online at www.liebertpub.com/tec

The four methods investigated for the analysis of accessible transport pathways through the pore space are illustrated in Figure 1, and described below. Quantitative analysis was carried out using the Skyscan software CTAn. All measurements were taken from a 1 mm^3^ region of interest (ROI) for each scaffold unless specified.

*A. Flood Fill:* This technique measures the size of the largest continuous pore space volume in the structure, relative to total pore volume.^11^ A 3D sweep of the Micro-CT dataset using the “Despeckle” function in CTAn was used to remove any pore space voxels disconnected from this continuous volume, and the volume of pore space remaining was measured. The Fiji Volume Viewer plugin was used to visualize the 3D object corresponding to this pore space volume, using a Flood Fill to color all connected pore space voxels.
*B. 3D Shrink-Wrap*: This method, based on the method by Fostad *et al.*,^12^ uses the CTAn function “ROI Shrink-Wrap.” This identifies the inaccessible regions of a scaffold ROI, according to a given definition of the minimum size of an inter-pore connection. This is analogous to identification of the accessible pore space for a virtual object, with diameter equal to this minimum connection size. The difference between the original ROI volume and the volume after Shrink-Wrap corresponds to the pore space that is accessible to this virtual object from the scaffold surfaces. For each diameter, % interconnectivity is defined by the following expression:
  \begin{align*}Interconnectivity \ ( \% ) = { \frac { V - V_s
}  { V - V_m } } \times \ 100 \tag { 1 } \end{align*}
where *V* is the total volume of the ROI, *V_s_* is the inaccessible scaffold volume after Shrink-Wrap, and *V_m_* is the volume of solid material (collagen) within the ROI. The minimum connection size was varied between 2 and 16 voxels, corresponding to a virtual object with diameter between 7.5 and 60 μm. The accessible volume (*V* − *V_s_*) was measured for each minimum connection size, to assess % interconnectivity as a function of virtual object diameter.
*C. Directional Shrink-Wrap:* This procedure is identical to the 3D Shrink-Wrap procedure, except that on selection of the ROI in CTAn, all surfaces but one of the Micro-CT dataset are artificially enclosed by inaccessible (pore wall) voxels. The accessible pore space therefore corresponds to the accessible volume for an object traveling from one specific scaffold surface.
% Interconnectivity as defined in equation (1) may therefore be assessed in different directions through the scaffold, providing a description of anisotropy.
*D. Percolation Diameter:* The Directional Shrink-Wrap technique also allows more detailed analysis of the transport pathways through the structure, in terms of the relationship between virtual object diameter, *d*, and maximum accessible distance from a chosen surface, *L*. According to percolation theory,^13,14^ this relationship may be written as
  \begin{align*}L\, \propto\, ( d - {d_c} ) ^{- 0.88} \tag{2}\end{align*}
The relationship between *L* and *d* is therefore controlled by the value of *d_c_*, and a constant of proportionality. Manipulation of equation (2) reveals that *d_c_* represents the size of the sphere that may travel through a scaffold of infinite dimensions, that is, as *L* approaches infinite values. This diameter will be referred to within the text as the percolation diameter. Plotting *d* as a function of *L*^−1/0.88^ allows calculation of *d_c_* as the intercept of the resulting plot.

### Cell culture and microscopy

Human periodontal ligament fibroblasts (Lonza) were cultured in high glucose Dulbecco's modified Eagle medium (LifeTechnologies) supplemented with 5% fetal bovine serum and 1% penicillin/streptomycin. The cells were detached at sub-confluence with trypsin-EDTA. Scaffold samples ∼10 × 10 × 2 mm were sterilized in 70% ethanol, before washing twice in phosphate-buffered saline (PBS; LifeTechnologies) and prewetting in medium. Excess medium was aspirated from the scaffolds before seeding onto the square face at a concentration of 64,000 cells in 50 μL medium per scaffold. Extra medium was added after 1 h at room temperature. Culture conditions were maintained at 37°C, 5% CO_2_, and humid atmosphere for 7 days, with medium changed three times per week.

At day 7, medium was removed and the scaffolds were washed in PBS, fixed with 10% formalin (Sigma-Aldrich), and permeabilized with a 10 min incubation in 0.1% Triton X-100/PBS (Sigma-Aldrich). Scaffolds were washed in PBS at each intermediate step. Cytoskeletal actin staining was performed with Alexa Fluor^®^ 488 Phalloidin (MolecularProbes) at 2.5 μL/200 μL in 1% bovine serum albumin/PBS (Sigma-Aldrich). Additionally, the cell nuclei were stained using a 1:2000 dilution of DAPI in PBS (MolecularProbes). Scaffolds were embedded in blocks of 15% gelatin/PBS (BioGel), and fixed with 10% formalin, allowing sectioning with a Leica VT1000 S Vibratome at a thickness of 200 μm to reveal the scaffold cross section.

Sections were imaged using a Yokogawa CV1000 Cell Voyager confocal microscope, which recorded the maximum fluorescent intensity over 11 z-slices, spacing 20 μm, for each scaffold cross section. Invasion distance within each scaffold was quantified by measurement of median cell position, as previously described.^8^ The mean and standard error of three measurements per scaffold are displayed.

### Statistical analysis

Statistical significance was tested using one-way ANOVA, followed by the Tukey-HSD *post hoc* test (*p* < 0.05).

## Results

### Scaffold morphology

**FIG. 2.**
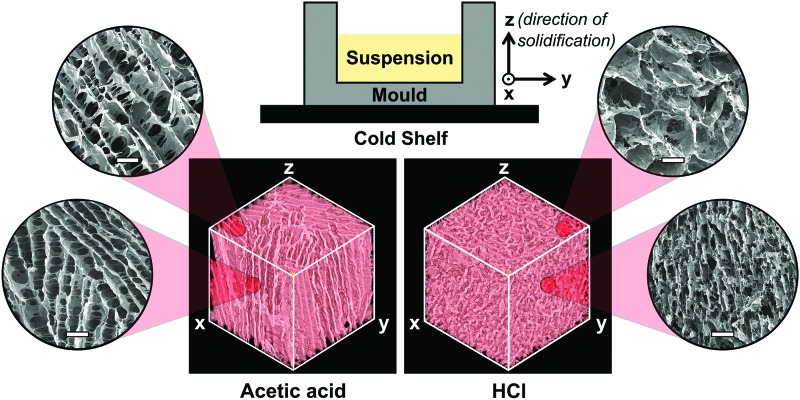
Micro-CT images (1 mm^3^) for the scaffolds fabricated with each suspension medium, along with SEM images of surface morphology. The schematic (*top*) defines the axis labels according to the direction of solidification during freeze-drying, that is, the *z*-axis. SEM scale bar is 100 μm. CT, computed tomography; SEM, scanning electron microscopy. Color images available online at www.liebertpub.com/tec

Control of the acid used for collagen suspension produced scaffolds with distinct structural variations, as shown in Figure 2. Although the pore shape clearly differs between the two scaffolds, the pore size remains relatively constant as previously reported.^8^ From the SEM images, it is apparent that both structures contain some anisotropy, with pore walls primarily oriented along the *z*-axis, that is, the direction of solidification during freeze-drying. Dark voids in the pore walls corresponding to inter-pore connections may be seen in all SEM images, but these are most apparent in the scaffold fabricated with acetic acid. Micro-CT elucidates the difference in pore shape between the two scaffolds, with plate-like pores resulting from the use of acetic acid, but equiaxed pores resulting from the use of HCl.

### Method A: Flood Fill

**FIG. 3.**
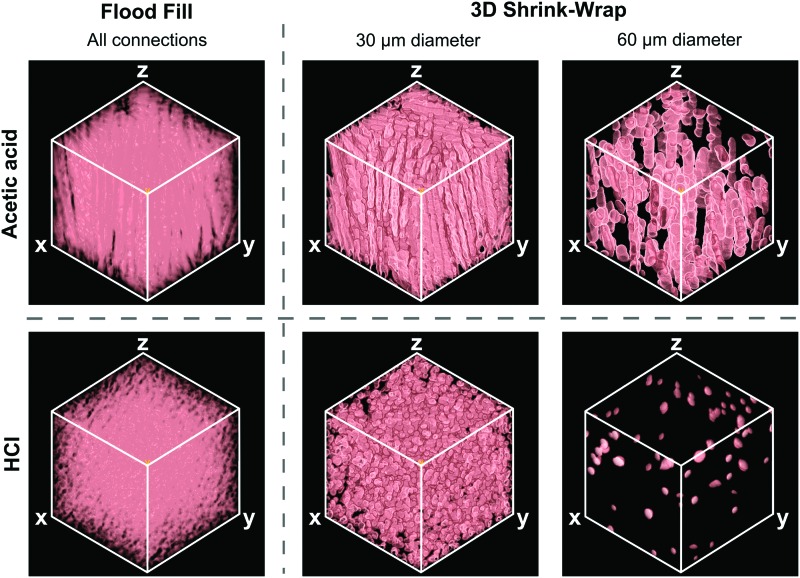
3D visualization of the accessible pore space within each scaffold, as measured by the Flood Fill and 3D Shrink-Wrap methods. Flood Fill displays the largest continuous pore space volume, while 3D Shrink-Wrap identifies the accessible pore space to a virtual object of variable diameter, by means of a user-defined minimum connection size. Each cube represents 1 mm^3^. Color images available online at www.liebertpub.com/tec

Figure 3 shows the largest continuous pore space volume for each scaffold, as evaluated by the Flood Fill technique. In both scaffolds, this volume extended throughout the entire structure. Quantitative analysis revealed that the proportion of pore space voxels connected to this continuous path is above 99% for both scaffolds. The gaps visible in Figure 3 therefore correspond almost entirely to the positions of the pore walls. Therefore, by this method of analysis, both scaffolds exhibit near-perfect pore accessibility.

### Method B: 3D Shrink-Wrap

Figure 3 also displays typical results from 3D Shrink-Wrap analysis, showing the pore space accessible to objects of 30 and 60 μm in diameter. 3D visualization reveals that the volume of accessible pore space is smaller in both scaffolds for the object of larger diameter. This difference is most apparent in the scaffold fabricated with HCl, in which the accessible pore volume for the 60 μm diameter object is restricted to just a few pores close to the scaffold surfaces. Therefore, by this method of analysis, % interconnectivity in 3D is enhanced by the use of acetic acid. No information on the anisotropy of this accessible pore space is available, however.

### Method C: Directional Shrink-Wrap

**FIG. 4.**
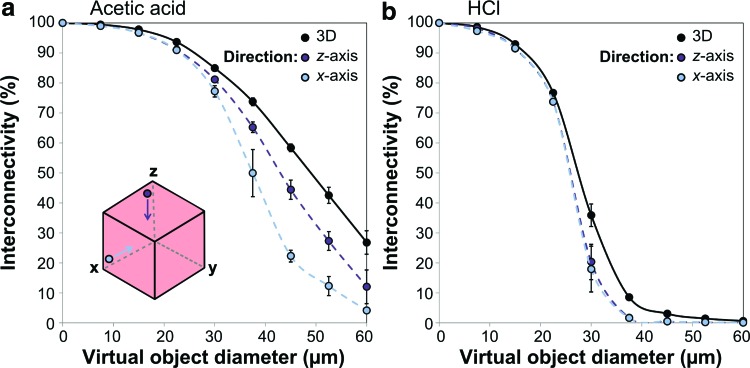
Plots of % interconnectivity against virtual object diameter, showing the effect of restricting the accessible surfaces of Micro-CT sub-volumes sampled from the scaffold fabricated with **(a)** 0.05 M acetic acid, or **(b)** 0.001 M HCl. This approach provides measurements of % interconnectivity, defined as the proportion of accessible pore space for a virtual object traveling from the outside of a 1 mm^3^ Micro-CT dataset, in specified directions as indicated in the schematic. Mean and standard error of the measurements from three different sub-volumes are displayed. Color images available online at www.liebertpub.com/tec

Figure 4 displays plots of % interconnectivity, as defined in equation (1), as a function of virtual object diameter. Many of these curves have a sigmoidal form, with values close to 100% for the smaller diameters as described above, followed by a steep drop to lower values at higher diameters. The curves for the scaffold fabricated with HCl show the most dramatic drop in % interconnectivity, both for the 3D measurements and for the directional measurements. The same Micro-CT sub-volumes were used for all three measurement methods in each of plots (a) and (b), demonstrating the influence of chosen direction through the scaffold on the observed results. The use of acetic acid produces much more direction-dependence in % interconnectivity, particularly at intermediate diameters, where the % interconnectivity along the *z*-axis is almost double that along the *x*-axis. However, all three curves for this scaffold are higher than any of the curves for the scaffold fabricated with HCl. Therefore, by this measurement method, the transport characteristics of the pore space appear to be enhanced in all directions by the use of acetic acid.

**FIG. 5.**
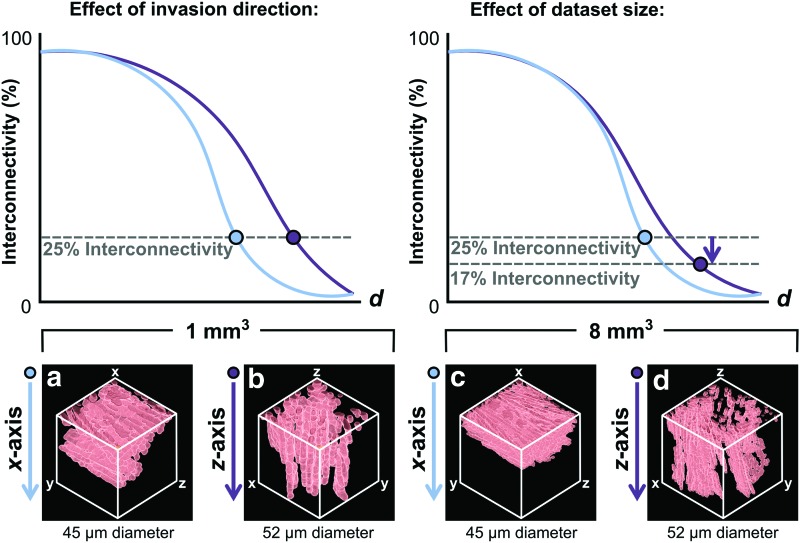
3D visualization of the results from Directional Shrink-Wrap of the scaffold fabricated with acetic acid, for comparison with the measured values of % interconnectivity. At a constant value of 25% interconnectivity, the corresponding accessible pore space could **(a)** be restricted to the scaffold surface, or **(b)** provide a continuous pathway from one side of the scaffold to the other. The possible effects of increasing the Micro-CT dataset size are also shown, with a higher % interconnectivity measured from **(c)** than from **(d)**, despite the considerably greater maximum accessible distance apparent in **(d)**. Color images available online at www.liebertpub.com/tec

A limitation of these plots is that they do not indicate how far the object of interest may travel in each given direction. This can hide important differences in the arrangement of the accessible pore space pathways. Figure 5 displays the accessible pathways corresponding to a measurement of 25% interconnectivity, in each direction through the scaffold fabricated with acetic acid. It is clear that a constant % interconnectivity measurement does not guarantee that an object may travel a constant distance through the scaffold, since in this case a continuous pathway along the whole length of the dataset only exists along the *z*-axis. Even more crucially, a lower % interconnectivity measurement can actually correspond to a greater accessible distance, as shown in Figure 5(d). On increasing the size of the Micro-CT dataset from 1 to 8 mm^3^, the % interconnectivity measured along the *z*-axis decreases to 17%, despite the continued existence of a continuous accessible pathway from one side of the dataset to the other. This result emphasizes the limitations of describing scaffold transport pathways in terms of % interconnectivity, as well as, indicating the importance of measurement length scale.

### Method D: percolation diameter

**FIG. 6.**
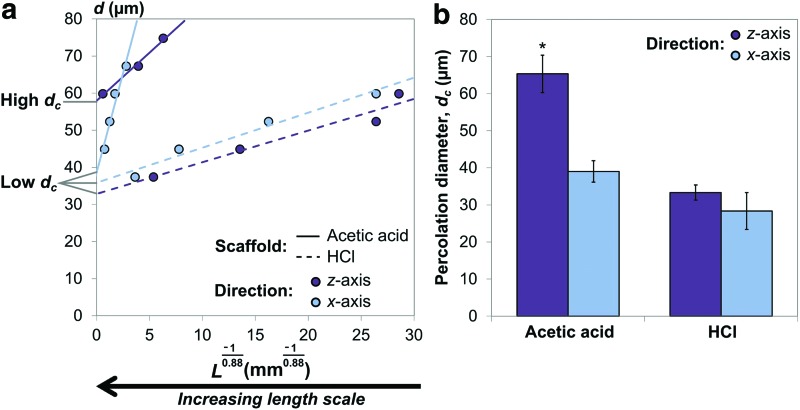
Application of percolation scaling principles to measurements of accessible distance, *L*, as a function of virtual object diameter, *d*. Plotting *d* against *L*^−1/0.88^ provides a measurement of the largest virtual object that could traverse a scaffold of infinite dimensions, given by the intercept, *d_c_*. Measurements for each condition are given as **(a)** representative examples of the corresponding plots, and **(b)** as the mean and standard error of three *d_c_* measurements for each condition. *Indicates statistical significance relative to all other conditions (*p* < 0.05). Color images available online at www.liebertpub.com/tec

Figure 6 shows the maximum distance accessible from each scaffold surface, *L*, plotted as a graph of virtual object diameter *d* against *L*^−1/0.88^ according to the relationship in equation (2). This approach allows calculation of the y-intercept, termed the percolation diameter, *d_c_*, which represents the size of the object that can travel through a scaffold of infinite dimensions. To allow measurements to be taken over a wider range of *L* values, the Micro-CT dataset sizes chosen for this analysis were increased to 8 mm^3^ as shown in Figure 5. The plots in Figure 6 indicate that the measurements taken from each scaffold follow a relationship close to that expected from equation (2), that is, a linear relationship between *L*^−1/0.88^ and *d*. The gradients and *d_c_* values extrapolated from these plots clearly differ according to suspension composition, but only differ significantly according to direction in the scaffold fabricated with acetic acid. This indicates that the use of HCl, in this case, produced scaffolds with an isotropic arrangement of transport pathways. However, the *d_c_* value for *z*-axis travel through the acetic acid scaffold is almost double any of the other values, indicating that the transport pathway arrangement within this scaffold is highly anisotropic.

The gradients of the plots for this scaffold are also relatively high, particularly for travel along the scaffold *x*-axis. This indicates that measurements of the transport pathway characteristics in this direction are highly sensitive to length scale. However, when extrapolated to infinite length scales, the characteristic transport pathway diameter, parameterized in terms of *d_c_,* showed no significant difference to those of the scaffold fabricated with HCl, as shown in Figure 6(b), with mean values in the range 30–40 μm.

### Fibroblast invasion and distribution

**FIG. 7.**
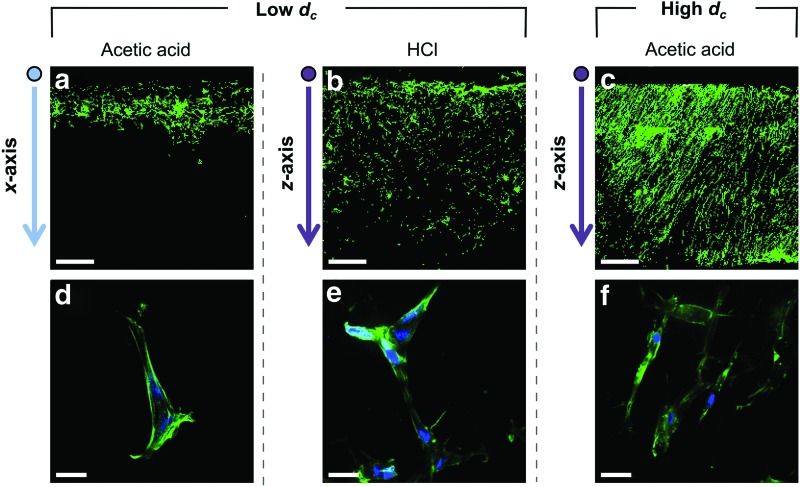
Fluorescent microscopy images showing fibroblast distribution (*top*, scale bar 500 μm) and morphology (*bottom*, scale bar 25 μm) at day 7, in response to invasion direction through the scaffold: **(a, d)** *x*-axis invasion, *low d_c_*, **(b, e)** *z*-axis invasion, *low d_c_*, **(c, f)** *z*-axis invasion, *high d_c_*. Color images available online at www.liebertpub.com/tec

The cross-sectional images in Figure 7 show the results of fibroblast invasion after 7 days culture, as a function of invasion direction and *d_c_*. The fibroblasts are clearly the most evenly distributed for invasion along the *z*-axis of the scaffold fabricated with acetic acid. Apart from containing a high *d_c_* value, invasion along the *z*-axis may be promoted by collagen contact guidance, resulting from the high pore wall alignment visible in Figure 2. Examination of *z*-axis invasion in the scaffold fabricated with HCl allowed decoupling of the dual effects of *d_c_* and invasion direction. As shown in Figure 7, the maximum invasion distance achieved along the *z*-axis is comparable in both scaffolds. However, the median invasion distance was measured to be only 0.71 ± 0.02 mm at low *d_c_*, compared with 1.02 ± 0.11 mm at high *d_c_*. This indicates that the fibroblasts are much less evenly distributed across the cross section of the low *d_c_* scaffold.

Invasion along the *x*-axis of the scaffold fabricated with acetic acid, which corresponds to low *d_c_* and low contact guidance, produced the lowest extent of fibroblast infiltration, with a median invasion distance of only 0.28 ± 0.05 mm. Importantly, high magnification imaging identified cells with an elongate morphology in all scaffolds, indicating fibroblast attachment and migration along the pore walls. These cell invasion results have therefore allowed the effects of pore wall orientation (estimated visually from SEM) and pore space anisotropy (reflected in the measurements of *d_c_*) to be distinguished, and revealed that both parameters are important in determining the distribution achieved by the cells.

## Discussion

Since the pore space of a freeze-dried collagen scaffold is a replica of the continuous ice crystal network formed during freeze-drying,^15^ continuous transport pathways through the structure are intuitively expected. The transport characteristics measured from these scaffolds are, however, clearly dependent on the chosen measurement technique. For instance, the SEM images in Figure 2 reveal the importance of scaffold characterization in 3D, ensuring that any anisotropy in pore size and orientation is accounted for. Additionally, although individual inter-pore connections can be identified from SEM images, they provide no information on the continuity of transport pathways through the structure.

As measured by the Flood Fill technique, both scaffolds appeared to be over 99% interconnected. Since this technique identifies the largest continuous pore space volume, this measurement approach is relevant for the diffusion of oxygen and nutrients through the bulk of the scaffold. However, it provides no information on how the available transport pathways vary when the movement of larger objects, such as cells, is considered. 3D Shrink-Wrap, on the other hand, provides distinct measurements of the pore space accessible from the scaffold surfaces according to the size of the object of interest. It is important to note that no matter how tortuous or complicated the pore space pathway, it will still be identified as accessible by the Shrink-Wrap method, so long as it is wider than the chosen virtual object diameter at all points.

The results in Figure 3 show that the accessible pore volume decreases as the size of the virtual object is increased. Importantly, the magnitude of this decrease is clearly dependent on scaffold structure, with the most dramatic changes observed in the scaffold fabricated with HCl. This technique can therefore identify differences in the transport properties of scaffolds that appear identical when evaluated by the Flood Fill technique. The Directional Shrink-Wrap goes a step further, and allows study of % interconnectivity as a function of direction. This is highly relevant for the biological scenario in which cells are invading from one specific face of the scaffold. Furthermore, as shown in Figure 4, a directional approach is vital for identifying any anisotropy in the arrangement of transport pathways through the pore space. In this case, this effect was only found to be substantial in the scaffold fabricated with acetic acid, despite the anisotropic appearance observed for both scaffolds under SEM.

However, as demonstrated by Figure 5, there are considerable limitations to the measurement of % interconnectivity in this way. First, a constant % interconnectivity can correspond to dramatic differences in the maximum accessible distance, depending on scaffold anisotropy. Second, there is a dependence between measured % interconnectivity and the choice of Micro-CT dataset size. This second limitation arises due to the intrinsic relationship between maximum accessible distance, *L*, and object diameter, *d*, described by equation (2). For small object diameters, *L* will be much larger than the dataset size, so the measured % interconnectivity is high. However, as the object increases in size, *L* will approach the size of the dataset, until eventually there is no continuous pathway that can transport the object from one side to the other. According to percolation theory, the apparent connectivity of the structure will be highly sensitive to dataset size during this transition region.^13^ This is therefore the cause of the dependence between measured % interconnectivity and dataset size observed in Figure 5, as well as the dramatic drop in measured % interconnectivity at intermediate connection sizes.

**FIG. 8.**
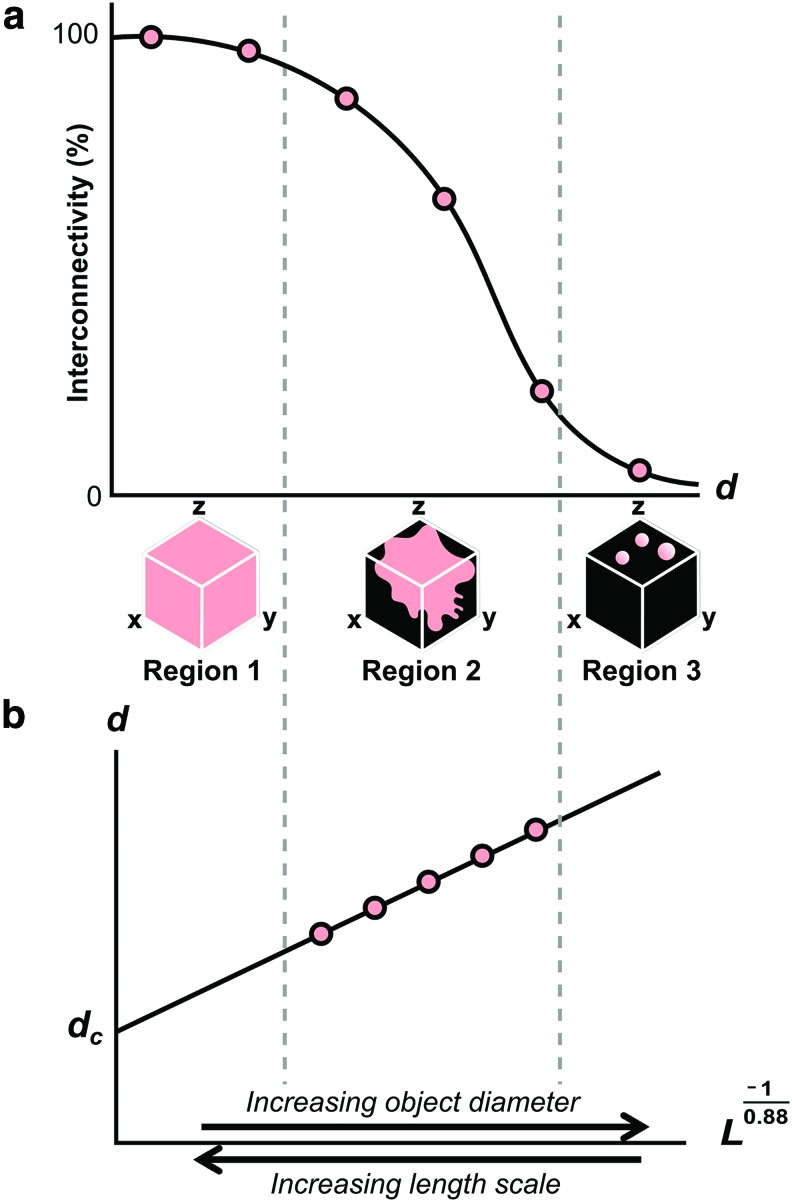
Schematic illustrating the relationship between **(a)** % interconnectivity plotted against virtual object diameter, *d*, and **(b)** *d* plotted against *L*^−1/0.88^, where *L* is the maximum accessible distance to an object of diameter *d*. In Region 1, *L* is larger than the Micro-CT dataset; therefore, the pore space appears fully interconnected. In Region 2, *L* approaches the dataset size, producing dramatic changes in % interconnectivity according to virtual object diameter, until Region 3, where only the pores at the scaffold surface are accessible. As shown in **(b)**, measurements from Region 2 can be used to identify the largest object diameter that can travel through the entire scaffold even at infinite length scales: the percolation diameter, *d_c_*. Color images available online at www.liebertpub.com/tec

Both limitations can be overcome, however, by direct measurement of the maximum accessible distance, and plotting according to the relationship in equation (2). Figure 8 illustrates how the result relates to the original % interconnectivity plot. Although both plots reveal a decrease in accessible pore space as the object diameter increases, in Figure 8b the transport pathways through the structure are quantified in terms of the accessible distance for a given diameter, rather than accessible volume fraction. For this reason, measurements are limited to object diameters for which the accessible pore volume is smaller than the dataset size. The maximum accessible distance for smaller objects may be found by extrapolation of these measurements to larger length scales, as shown in the Figure. This allows identification of the largest object diameter that can travel through a scaffold of infinite dimensions, given by the intercept, *d_c_*. This parameter, termed the percolation diameter, describes the characteristic size of the transport pathways through the structure.^14,16^

Calculation of *d_c_* requires a series of *L* and *d* measurements that are representative of the entire scaffold in all dimensions.^17^ If the object diameter is too large, its accessible distance will be too small to be representative of the structure as a whole.^13^ However, when the object diameter is too small, the accessible distance diverges to infinity, as described by equation (2). The choice of Micro-CT dataset size is vital in ensuring that enough measurements can be made between these two extreme cases. For this reason, the Micro-CT dataset sizes used to obtain the results in Figure 6 were increased from 1 to 8 mm^3^.

The gradients of the plots in Figure 6 relate to the smallest representative volume, or repeat length, of each structure, which determines the sensitivity of these measurements to changes in length scale.^13^ In this case, the highest gradient corresponds to the *x*-axis of the scaffold fabricated with acetic acid. Therefore, the main transport pathways through the structure will appear to have a greater diameter when viewed at low length scales, rapidly decreasing as the length scale of interest is increased. In fact, the results in Figure 6(b) show that the characteristic diameter of the transport pathways measured along the *x*-axis in the limit of infinite system size, as described by *d_c_*, is not significantly different from either direction in the scaffold fabricated from HCl. This contrasts with the results from the plots in Figure 4, which indicate that acetic acid produces higher % interconnectivity than HCl in all directions. Consideration of percolation theory is therefore vital in determining how applicable the measurements made from finite Micro-CT datasets are to the properties of bulk samples.

As shown in Figure 6(b), many of the measured *d_c_* values are close to the diameter of a single cell, that is, around 30 μm.^18^ Only the *z*-axis of the scaffold fabricated with acetic acid has a significantly higher *d_c_* value, which indicates that it may offer considerably less resistance to cell invasion. This was tested by examination of fibroblast invasion as a function of direction through the scaffold fabricated with acetic acid. The relative influence of pore wall alignment and transport pathway characteristics could also be assessed, by examining *z*-axis invasion in the scaffold fabricated with HCl, which contains a low *d_c_* value while maintaining high pore wall alignment in the direction of invasion, as shown in Figure 2. Although the high pore wall alignment increased the maximum invasion distance achieved by some of the cells, the overall cell distribution was visibly improved in the scaffold with higher *d_c_*. This result shows that quantitative parameterization of the transport pathways through the pore space is vital for prediction of cell response to scaffold structure. It also emphasizes the inadequacy of the Flood Fill and 3D Shrink-Wrap techniques, since the % interconnectivity of the two scaffolds appeared identical by the first method, and no differences according to direction could be detected by either method.

Since tissue ingrowth into a biomaterial scaffold may require pore connections of up to 100 μm, it is important to provide a scalable analysis of pore accessibility at such large diameters.^19^ The strength of the *d_c_* measurement approach is that it accounts for changes in these measurements according to the length scale of interest, while providing a ranking of the transport characteristics at high length scales. Such image-based analysis methods also have the advantage that they are capable of detecting all possible transport pathways within a given field of view, and are not limited by the lack of infiltration efficiency that may exist in experimental analogues, such as the movement of spheres of known sizes. However, a comparison between the numerical methods presented here and physical properties measured by the diffusion of fluorescent molecules, for instance, would be an interesting topic for further study.

An important consideration is that cells can deform themselves and their environment in response to physical barriers, both mechanically and by matrix proteolysis. Measurements of *d_c_* should not, therefore, be considered an absolute limit to the size of the cell that can access the structure, but rather a description of the structural resistance to cell invasion. Importantly, this approach allows quantitative parameterization of the scaffold transport pathways in combination with other structural features, enabling a more effective assessment of their relative importance in determining cell behavior. As proof of principle, we have demonstrated the independence of pore wall alignment and *d_c_* in determining cell behavior, and we have shown that both parameters must be optimized to maximize the extent of fibroblast invasion. In recent work, we have also used this method to indicate the existence of a critical *d_c_* threshold for fibroblast invasion in collagen scaffolds of constant pore size.^8^ This methodology therefore contains exciting potential for identification of the key structural criteria for application-based scaffold design.

## Conclusions

By investigating a range of Micro-CT characterization methods, in combination with the demonstrative example of fibroblast invasion, we have identified the key considerations for assessment of the transport characteristics of porous scaffolds. First, the accessible pathways for objects of variable diameter must be considered, to predict the amount of resistance to cellular invasion. Second, their characteristics must be measured as a function of direction, to identify any structural anisotropy in the pore space pathways. Finally, we have shown that measurements of % interconnectivity are sensitive to the chosen dimensions of a Micro-CT dataset. Using principles from percolation theory, transport characteristics can instead be parameterized in terms of the characteristic size of the pathways encountered by an invading object: the percolation diameter, *d_c_*. Along with providing quantitative assessment of the pathways available over a range of length scales, this approach has shown potential for independent correlation between transport characteristics, biological response, and further properties such as pore wall alignment, for an enhanced understanding of the ways in which each parameter may be used to influence cell behavior.
